# Carl Blomqvist, Nordic network builder in oncology (1951–2023) †

**DOI:** 10.2340/1651-226X.2024.35090

**Published:** 2024-02-02

**Authors:** Micaela Hernberg, Johanna Mattson, Mikko Tenhunen, Jonas Bergh, Johan Ahlgren, Lars Holmberg, Malin Sund, Mef Nilbert

**Affiliations:** a Helsinki Comprehensive Cancer Centre, Finland; b Karolinska Comprehensive Cancer and University Hospital, Sweden; c Karolinska institutet, Sweden; d Regional Cancer Centre mid-Sweden, Sweden; e Uppsala University, Sweden; f University of Helsinki and Helsinki University Hospital; g Umeå University, Sweden; h Skane University Hospital Comprehensive Cancer Centre, Sweden; i Lund University, Sweden

**Figure:**
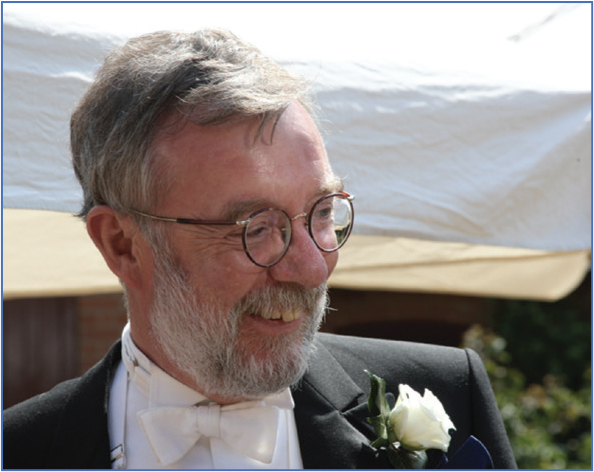


On 27 November 2023, the Nordic oncology community lost a strong network scientist when Professor Carl Blomqvist passed away at the age of 72 after a short period of illness.

Carl ‘Calle’ Blomqvist was a role model who inspired and educated a whole generation of oncologists in Finland and beyond. Following a PhD on skeletal metastases from breast cancer, he performed clinical and translational studies in the fields of breast cancer, bone and soft tissue sarcoma, and testicular cancer. During a long scientific career, Calle published more than 450 scientific articles and supervised 19 PhD projects, in Finland and in Sweden. His scientific contributions range from biomarker studies to clinical breast cancer trials that contributed to the current treatment standards and administration schemes. Indeed, he pursued clinical duties at the Helsinki University Hospital Comprehensive Cancer Center until his recent 72nd birthday and as late as this fall some of us were scrutinizing figures with Calle in on-going projects.

Born in Solna, Sweden, the family moved back to Finland when Calle was 3 years old. He graduated from Vasa Svenska Lyceum in 1970, completed studies in medicine at the University of Helsinki in 1976, obtained a specialization in medical oncology and radiotherapy in 1985 and successfully defended his PhD in 1988.

Calle worked as an adjunct professor and professor of oncology at the University of Helsinki and as the chief physician of the outpatient department of oncology. He was recruited to Sweden where he worked as senior consultant, responsible for breast cancer and sarcoma, at the Department of Oncology, Uppsala University Hospital 1999–2010 linked to an adjunct professorship and later held a part-time position as senior consultant in oncology at the Örebro University Hospital.

Throughout his career, Calle developed and maintained close collaborations within the Nordic professional networks. He was a true ambassador for Nordic collaboration and his engagement secured Finnish participation in joint Nordic initiatives such as joint research studies and clinical trials, particularly within the fields of breast cancer and sarcoma. No distance was too far to travel for such research collaboration, and Calle was actively involved in both local and virtual research groups until his too early death.

Calle was an active reviewer for *Acta Oncologica*. His contributions were characterized by high quality, respect for timelines, constructive criticism, and attention to detail. In 2018, he joined the Acta Oncologica board and in this role was an advocate for the development of the journal and showed strong support for the transition to Open Access.

Calle mastered the art of medicine in parallel with the science of medicine. As a professional, he was highly regarded among patients and colleagues. Many colleagues in Finland and Sweden have had the opportunity to profit from Calle’s expertise and broad clinical skills in oncology. His empathic encounters with patients, combined with exquisite scientific thinking, granted his patients the best possible treatments. Calle was highly appreciated by his clinical colleagues for his knowledge and his collateral thinking in the management  of patients in circumstances when there was no evidence basis to guide treatment recommendations. As a scientist and expert, he was creative, intelligent, thorough, and followed the highest professional standards. He showed generosity toward younger colleagues and provided hands-on support and mentoring in specific tasks as well as long-term career advice.

As a person, Calle was a man of honor. He was modest, constructive and positive. He was honest and trustworthy. He was a thinker and a doer. According to his wife, Liisa, innovation flourished when he could enjoy gardening or fishing at their country paradise in Lohja outside of Helsinki or when sailing in the Finnish archipelago. His beloved family was his greatest priority, and his role as grandfather was of highest value and importance for him.

We express our sincere condolences and sympathy to Calle’s family, his wife Professor emerita Liisa Hakamies-Blomqvist, his son Oliver and his daughter Sofia and their families for the loss of a dear husband, father, father-in-law, and grandfather.

Calle Blomqvist was a distinguished professional and researcher who will remain among the most prominent leaders in our Nordic oncological community. We mourn his passing. We learned so much from him. We remember Calle with the highest respect and gratitude.

